# Phenolic Content and Antioxidant Activity in Apples of the ‘Galaval’ Cultivar Grown on 17 Different Rootstocks

**DOI:** 10.3390/antiox11020266

**Published:** 2022-01-28

**Authors:** Aurita Butkeviciute, Vytautas Abukauskas, Valdimaras Janulis, Darius Kviklys

**Affiliations:** 1 Department of Pharmacognosy, Lithuanian University of Health Sciences, Sukileliu Ave. 13, LT-50162 Kaunas, Lithuania; valdimaras.janulis@lsmuni.lt; 2 Laboratory of Biochemistry and Technology, Lithuanian Institute of Horticulture, Lithuanian Research Centre for Agriculture and Forestry, Kauno Str. 30, LT-54333 Babtai, Lithuania; vytautas.abukauskas@lammc.lt (V.A.); darius.kviklys@lammc.lt (D.K.); 3 Norwegian Institute of Bioeconomy Research—NIBIO Ullensvang, Postboks Str. 11, NO-1431 Ås Lofthus, Norway

**Keywords:** antioxidants, food quality, *Malus*, polyphenol

## Abstract

Apple cultivars are one of the main factors setting the composition of bioactive compounds in apples and the quality of the fruit. However, research has been providing increasing amounts of data on the influence of rootstocks on the variations in the composition of bioactive compounds in apples. The aim of the study was to determine the influence of rootstocks on the changes in the qualitative and quantitative composition of phenolic compounds and their antioxidant activity in vitro in apple flesh and peel. HPLC analyses of phenolic compounds in apple samples were performed. The rootstock–scion combination had a significant effect on the composition and antioxidant activity of phenolic compounds in apple samples. Depending on the rootstock, the total content of phenolic compounds in apple flesh of the ‘Galaval’ cultivar could vary by 2.9 times, and in the peel by up to 90%. The genotype of the rootstock resulted in the highest variation in total flavan-3-ol content in apple flesh—by as much as 4.3 times—while the total content of flavonols varied by 2.1 times. In apple peel, on the contrary, the greatest variation was recorded for the total flavonol content (by 4.4 times), and the total flavan-3-ol content varied the least (by 1.8 times). A proper match of a cultivar and a rootstock can program a fruit tree to grow larger amounts of higher-quality, antioxidant-rich, and high-nutrition-value fruit.

## 1. Introduction

Apples are among the most widely consumed fruits in the world. In 2020, the amount of apple fruit grown in the world reached 84.6 million tons [1]. Apples and their processed products are widely used in the production of food supplements, food industry products, and beverages [2]. Apples are an important component of the human food chain [3], and their nutritional properties are determined by a complex of biologically active phenolic and triterpenic compounds [2]. Phenolic compounds (hydroxycinnamic acids, flavan-3-ols, flavonols, dihydrochalcones, and anthocyanins) are the predominant group of secondary metabolites in apples [2,3], and the qualitative and quantitative composition of which in apple peel and flesh differs [4]. Phenolic compounds establish the antioxidant activity of apples [2,5]. Phenolic compounds, acting as reducing agents by donating hydrogen, quenching singlet oxygen, acting as chelators, and trapping free radicals, protect DNA, proteins, lipids, and other macromolecular structures from the damaging effects of free radicals [3]. Phenolic compounds lower blood glucose levels [6,7] and have anti-cancer [8], anti-inflammatory [9,10], antimicrobial [11,12], anti-obesity [13,14], cardioprotective [15,16], and neuroprotective [17] effects. Daily consumption of fruit rich in a complex of biologically active phenolic compounds reduces the risk of developing chronic diseases [5].

The qualitative and quantitative composition of phenolic compounds in apples varies depending on biotic and abiotic factors [18]. Agrotechnology of fruit tree cultivation, climatic conditions, soil composition, UV radiation, and the location of the fruit in the fruit tree crown. may influence changes in the content of phenolic compounds in apple fruit [4,19,20]. Apple cultivar is one of the main factors determining the composition of biologically active compounds in apples and the quality of the fruit [21]. However, scientific literature has been providing increasing amounts of data on the influence of rootstocks on the variation in the quantitative and qualitative composition of biologically active compounds in apples [22]. Rootstocks significantly affect the growth properties of fruit trees, the onset of fruiting, the yield, and resistance to frost and drought, and determine the organoleptic properties and mineral composition of the fruit [23,24,25,26,27,28]. Rootstocks have also been found to determine the qualitative and quantitative composition of phenolic compounds in apples [20,29]. A properly selected cultivar and rootstock can program the fruit tree to produce more high-quality, antioxidant-rich, and high-nutrition-value fruit [30,31]. In recent years, relatively high numbers of new apple rootstocks have been delivered by breeding centers around the world. These rootstocks outperform traditionally used rootstocks in productivity and resistance to various abiotic and biotic stresses. Scientific literature presents fragmentary research data on the influence of apple rootstocks on the qualitative and quantitative composition of phenolic compounds and antioxidant activity. For this reason, it is expedient to assess the variation in the qualitative and quantitative composition of individual phenolic compounds in apple flesh and peel samples and to identify apple rootstocks that determine the highest phenolic content in apples and the strongest antioxidant activity in vitro. The obtained data are important from a practical point of view, which allows for selecting the right combinations of cultivars and rootstocks, and providing consumers with higher yields of apples with known biologically active compounds as well as food and food supplements produced from those apples.

The aim of the study was to determine the influence of rootstocks on the changes in the qualitative and quantitative composition of phenolic compounds and their antioxidant activity in vitro in apple flesh and peel samples.

## 2. Materials and Methods

### 2.1. Plant Material

In this study, we evaluated the apple cultivar ‘Galaval’ grown with 17 rootstocks of different growth groups (Table 1). The orchard was planted at the Institute of Horticulture (Babtai, Lithuania), a branch of the Lithuanian Research Center for Agriculture and Forestry (coordinates: 55°60′ N, 23°48′ E) in spring 2017 in the frame of a multi-location apple rootstock trial led by the EUFRIN (European Fruit Research Institute Network) Apple & Pear Variety & Rootstock Testing Working Group. Planting distances depended on rootstock vigor: 3.5 × 1 for the dwarf rootstocks and 3.5 m × 1.5 m for the semi-dwarf rootstocks. Each rootstock was replicated four times, with three trees per plot. Trees were trained as slender spindles. A sustainable plant protection system was used for orchard management [32]. Apples of the ‘Galaval’ cultivar were harvested at optimal fruit maturity. The study was conducted during 2020–2021.

### 2.2. Chemicals and Solvents

All solvents, reagents, and standards used were of analytical grade. Acetonitrile and acetic acid were obtained from Sigma-Aldrich GmbH (Buchs, Switzerland) and ethanol from AB Stumbras (Kaunas, Lithuania). Hyperoside, rutin, quercitrin, phloridzin, procyanidin B1, procyanidin B2, and chlorogenic acid standards were purchased from Extrasynthese (Genay, France); reynoutrin, (+)-catechin, and (−)-epicatechin from Sigma-Aldrich GmbH (Steinheim, Germany); and avicularin, procyanidin C1, and isoquercitrin from Chromadex (Santa Ana, CA, USA). Purified deionized water used in the tests was prepared with the Milli-Q^®^ (Millipore, Bedford, MA, USA) water purification system.

The reagents used in antioxidant activity assays—6-hydroxy-2,5,7,8-tetramethylchroman-2-carboxylic acid (Trolox), 2,2-diphenyl-1-picrylhydrazyl (DPPH), and sodium acetate—were obtained from Scharlau (Barcelona, Spain), 2,4,6-tri(2-pyridyl)-s-triazine (TPTZ) was obtained from Carl Roth (Karlsruhe, Germany), and iron (III) chloride hexahydrate (FeCl_3_·6H_2_O) from Vaseline-Fabrik Rhenania (Bonn, Germany).

### 2.3. Preparation of Apple Lyophilizate and Apple Extracts

Apple flesh and peel samples were prepared as described by Butkeviciute et al. [33]. Extracts of apple flesh and peel samples were prepared as described by Butkeviciute et al. [34].

### 2.4. Evaluation of Phenolic Compounds by HPLC-PDA Method

Qualitative and quantitative high-performance liquid chromatography (HPLC) analyses of phenolic acid and flavonoids in the apple flesh and peel extracts were performed by applying the method described by Liaudanskas et al. [21].

### 2.5. Antioxidant Activity Assays

Antioxidant activities of the apple flesh and peel extracts were determined by two different antioxidant capacity assays; namely, in vitro spectrophotometric DPPH free radical scavenging and ferric (FRAP) reducing activity assays using a spectrophotometer (Spectronic CamSpec M550, Garforth, UK). To perform the DPPH^•^ free radical scavenging assay, 3 mL of DPPH^•^ solution was mixed with 10 μL of apple flesh and apple peel extracts. A decrease in absorbance was measured at λ = 517 nm [35]. The FRAP solution included TPTZ (0.01 M dissolved in 0.04 M HCl), FeCl_3_·6H_2_O (0.02 M in water), and an acetate buffer (0.3 M, pH 3.6) (ratio 1:1:10). During the analysis, 3 mL of a freshly produced FRAP reagent was mixed with 10 µL apple flesh and apple peel extracts. An increase in absorbance was measured at λ = 593 nm [36]. The calculation of the antioxidant activity of apple flesh and apple peel extracts was the following: the antioxidant activity of the extracts was calculated from the Trolox calibration curve and expressed as a μM Trolox equivalent (TE) per gram of absolutely dry weight (DW). TE was calculated according to the formula TE = c × V/m (μM/g), where c was the concentration of Trolox established from the calibration curve (in μM), V was the volume of the apple extract (in L), and m was the weight (precise) of the lyophilized apple powder (in g).

### 2.6. Statistical Analysis

The analysis of the data of the HPLC method was performed by using Microsoft Office Excel 121 (Microsoft, Redmond, WA, USA) and SPSS, version 25.0 (SPSS Inc., Chicago, IL, USA) software. All the results estimated during the HPLC analysis were presented as means of three consecutive test results and standard deviations. The univariate analysis of variance (ANOVA) was applied in order to establish whether the differences between the compared data were statistically significant. The hypothesis about the equality of variances was verified by applying Levine’s test. If the variances of independent variables were found to be equal, Tukey’s multiple comparison test was used. The differences were regarded as statistically significant at *p* < 0.05. The comparison of the chemical composition between the apple samples was carried out by applying the hierarchical cluster analysis, using the squared Euclidean distance.

## 3. Results and Discussion

### 3.1. Influence of Rootstocks on the Phenolic Content in Apple Flesh Samples

With the recent intensive development of horticulture, but at the same time declining area of land suitable for agriculture, new ways are being sought to increase the productivity of fruit plants to grow higher yields of high-quality, high-nutrition fruit. The application of rootstocks in modern horticulture is one of the main ways to achieve the above-listed properties. An appropriate matching of the apple cultivar and rootstock can initiate the synthesis of phenolic compounds and increase their content in the fruit [22]. Therefore, we conducted studies in which we evaluated the trends of the variation in the phenolic profiles and to identify the most promising apple rootstocks that ensure the highest content of individual phenolic compounds in apples.

Our study was performed on the ‘Galaval’ cultivar, which is one of color mutations of the ‘Gala’ cultivar. The ‘Gala’ cultivar and its clones are widely cultivated and are in the top five of the world’s apple cultivars. The comparison of the phenolic profiling of ‘Royal Gala’ with the other four main apple cultivars grown in Australia showed that it accumulated the highest content of phenolic acids and flavonoids [5], although the total phenolic content was average. The same was established in a study performed in Brazil, where ‘Gala’ was compared with ‘Fuji’ and ‘Golden Delicious’ cultivars [37].

**Figure 1:**
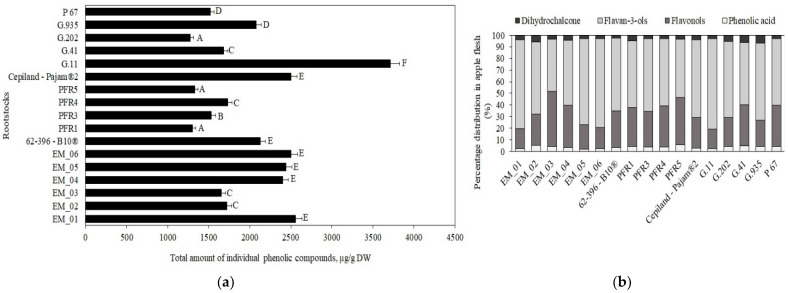
Influence of rootstocks on the quantitative composition of phenolic compounds: (**a**) Total variation in the number of phenolic compounds in apple flesh samples; (**b**) percentage distribution of phenolic compounds in apple flesh samples. The means followed by different letters are significantly different at *p* < 0.05.

In our study, the total phenolic content in apple flesh samples of the ‘Galaval’ cultivar ranged from 1278 μg/g ± 50 μg/g to 3715 μg/g ± 235 μg/g, depending on the rootstock (Figure 1, Panel a). The maximum total content of phenolic compounds was 3715 μg/g ± 235 μg/g when grown with the strong-dwarf rootstock G.11, and the minimum content was 1278 μg/g ± 50 μg/g with the semi-dwarf rootstock G.202 from the same breeding program in the USA (Figure 1, Panel a). Lauzike et al., studying the apple cultivar ‘Auksis’ on super-dwarf P 22 and dwarf P 60 rootstocks, found that the total content of phenolic compounds in apple samples ranged from 2470 μg/g to 3110 μg/g [30]. These research data confirm the results of our study.

The following compounds were identified and quantified in apple samples: chlorogenic acid, flavan-3-ols ((+)-catechin, (−)-epicatechin, procyanidin B1, procyanidin B2, and procyanidin C1), flavonols (rutin, hyperoside, isoquercitrin, reynoutrin, avicularin, and quercitrin), and phloridzin. The percentage distribution of the determined phenolic compounds in apple flesh samples depending on the rootstocks is shown in Figure 1, Panel b. Compounds of the flavan-3-ol group predominated in apple flesh samples, with rootstock distributions ranging from 45% to 78% of the total phenolic compounds identified and quantified.

**Figure 2:**
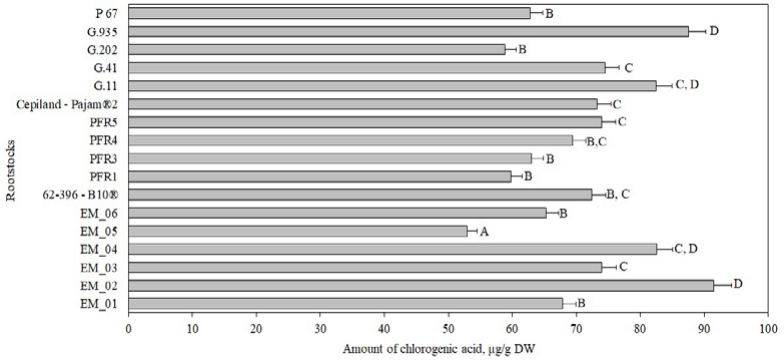
Influence of rootstocks on the quantitative composition of chlorogenic acid in apple flesh samples. The means followed by different letters are significantly different at *p* < 0.05.

Chlorogenic acid accounted for 2% to 6% of the total amount of phenolic compounds identified and quantified in the tested apple flesh samples (Figure 1, Panel b). The chlorogenic acid content in apple flesh samples, depending on the rootstock, ranged from 53 ± 2 μg/g to 92 ± 5 μg/g (Figure 2). The largest amount of chlorogenic acid (92 ± 5 μg/g) was found in flesh samples of apples grown on a small dwarf rootstock EM_02, although there were no significant differences from EM_04, G.11, or G.935 rootstocks. Meanwhile, the smallest amount of chlorogenic acid among all the tested rootstocks (53 ± 2 μg/g) was found in the group grown on the small dwarf rootstock EM_05 (Figure 2).

The amount of chlorogenic acid in apples depended on the genotype of the cultivar. The amount of chlorogenic acid in red-fleshed and white-fleshed apple samples was found to vary from 52 µg/g to 306 µg/g [38]. In our study, apple flesh samples of the ‘Galaval’ cultivar had a smaller amount of chlorogenic acid, compared to that (210 μg/g) found in apple flesh samples of the ‘Brookfield Gala’ cultivar [38]. Kiczorowski et al. in their study found that the chlorogenic acid content in apple flesh of the ‘Sampion’ cultivar grown on a semi-dwarf rootstock M.26 and dwarf rootstocks P 2, M.9, and P 22 varied from 69 μg/g to 124 μg/g [39]. The researchers found that the highest levels of chlorogenic acid were detected in flesh samples of apples grown on the dwarf rootstock M.9, and the lowest in flesh samples of apples grown on the semi-dwarf rootstock M.26. In our study, we obtained the opposite results after evaluating 17 rootstocks of different vigor and origin. This shows the importance of the rootstock–cultivar combination.

**Figure 3:**
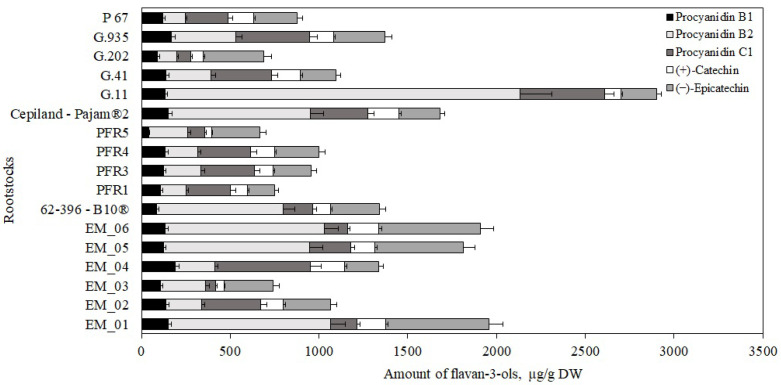
Influence of rootstocks on the quantitative composition of flavan-3-ols in apple flesh samples.

Flavan-3-ol compounds predominated in apple flesh samples. Our study showed that the total amount of the identified flavan-3-ol group compounds in ‘Galaval’ apples could differ by as much as several times depending on the rootstocks (Figure 3). The highest total amount of flavan-3-ols (2903 ± 126 μg/g) was found in apples grown on the strong-dwarf rootstock G.11, and the lowest (668 ± 32 μg/g) was found on apples grown on the dwarf rootstock PFR5 (Figure 3). Slatner et al. found that the flavan-3-ol content in apple fruit samples of the ‘Ligol’ cultivar grown on rootstocks M.9, M.26, and P 60 varied from 38 μg/g to 62 μg/g [40]. Bars-Cortina et al. studied apple flesh samples of the ‘Brookfield Gala’ cultivar and found a smaller amount of flavan-3-ols (197 µg/g) compared to that found in our study [38].

The amount of monomeric ((+)-catechin and (−)-epicatechin) flavan-3-ols in apple flesh samples varied. The highest (−)-epicatechin content (582 ± 16 μg/g) was found in flesh samples of apples grown on the semi-vigorous rootstock EM_01, and the lowest (154 ± 11 μg/g) in flesh samples of apples grown on the semi-dwarf rootstock PFR1 (Figure 3). The highest (+)-catechin content (191 ± 9 μg/g) was found in flesh samples of apples grown on the super dwarf rootstock EM_04, and the lowest (39 ± 2 μg/g) was found in flesh samples of apples grown on the dwarf rootstock PFR5 (Figure 3). Preti and Tarola found that the amount of (+)-catechin in flesh samples of ‘Royal Gala’ apples was 90 μg/g, and the amount of (−)-epicatechin was 95 μg/g [2]. In apple flesh samples of the ‘Galaval’ cultivar, we found higher levels of (+)-catechin and (−)-epicatechin compared to those detected by Preti and Tarola.

The oligomeric compounds of the flavan-3-ol group (procyanidin B1, procyanidin B2, and procyanidin C1) predominated in apple flesh samples. The results of the study by Belviso et al. showed that procyanidins were among the most widely detected flavan-3-ol compounds in apple samples [41]. Procyanidin B2 predominated among all the detected procyanidins in apple flesh samples of the ‘Galaval’ cultivar. The highest procyanidin B2 content (2000 ± 100 μg/g) was found in flesh samples of apples grown on the strong-dwarf rootstock G.11, and the lowest (106 ± 9 μg/g) was found in flesh samples of apples grown on the semi-dwarf rootstock G.202 (Figure 3). The amount of procyanidin B1 found in apple flesh samples was the lowest compared to the amounts of procyanidin B2 and procyanidin C1. The highest content of procyanidin B1 (170 ± 13 μg/g) was found in flesh samples of apples grown on the strong-dwarf rootstock G.935, and the lowest (42 ± 6 μg/g) in flesh samples of apples grown on the dwarf rootstock PFR5 (Figure 3). In 2017, Kviklys et al. studied apple samples of the ‘Auksis’ cultivar grown on dwarf rootstocks P 67 and B.396 and found higher levels of procyanidin B1 (respectively, 235 μg/g and 209 μg/g) compared to those we found in apple flesh samples of the ‘Galaval’ cultivar grown on rootstocks P 67 and 62-396-B10^®^ (respectively, 87 ± 4 μg/g and 119 ± 11 μg/g) [42]. The researchers also found higher levels of procyanidin B2 in apple samples of the ‘Auksis’ cultivar grown on dwarf rootstocks P 67 and B.396 (respectively, 1162 μg/g and 1099 μg/g) compared to those we found in apple flesh samples of the ‘Galaval’ cultivar grown on rootstocks P 67 and 62-396-B10^®^ (respectively, 127 ± 12 μg/g and 712 ± 17 μg/g) [42]. Different research results might have been influenced by the cultivar chosen for the analysis. Recent studies have shown that the apple cultivar may substantially influence the chemical properties of the fruit [43].

**Figure 4:**
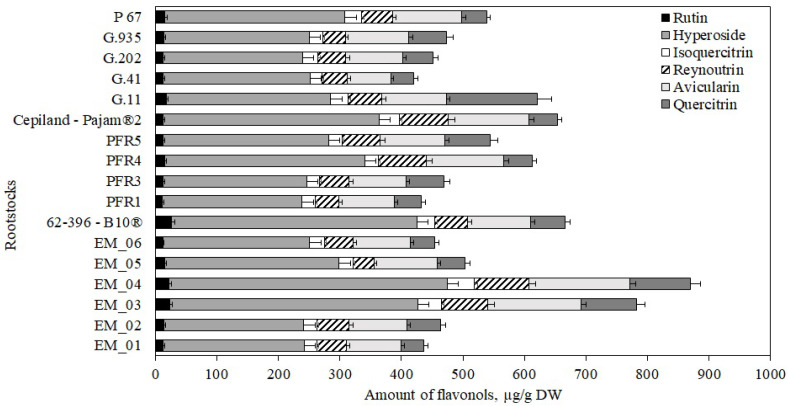
Influence of rootstocks on the quantitative composition of flavonols in apple flesh samples.

The following flavonols were found in apple flesh samples: rutin, hyperoside, isoquercitrin, reynoutrin, avicularin, and quercitrin. The highest total amount of flavonols (870 ± 35 μg/g) were found in apple flesh samples of the ‘Galaval’ cultivar grown on the super dwarf rootstock EM_04, and the lowest (421 ± 10 μg/g) was found in flesh samples of apples grown on the strong-dwarf rootstock G.41 (Figure 4). In 2014, Kviklys et al. studied samples of ‘Ligol’ apples grown on dwarf rootstocks P 67 and B.396 and found lower total flavonol levels (respectively, 321 μg/g and 340 μg/g) compared to those we found in apple flesh samples of the ‘Galaval’ cultivar grown on rootstocks P 67 and 62-396-B10^®^ (respectively, 538 ± 27 μg/g and 666 ± 20 μg/g) [22].

Hyperoside predominated among the identified and quantified flavonol group compounds. The highest levels of hyperoside, avicularin, reynoutrin, and isoquercitrin (accordingly, 453 ± 25 μg/g, 164 ± 11 μg/g, 89 ± 10 μg/g, and 44 ± 13 μg/g) were found in flesh samples of apples grown on the super dwarf rootstock EM_04. (Figure 4). Meanwhile, the largest amount of quercitrin (148 ± 18 μg/g) was found in flesh samples of apples grown on the strong-dwarf rootstock G.11 (Figure 4). Of all the flavonol compounds identified and quantified, the amount of rutin was found to be the lowest. The largest amount of rutin (26 ± 2 μg/g) was found in flesh samples of apples grown on the dwarf rootstock 62-396-B10^®^ (Figure 4). We found higher levels of hyperoside, isoquercitrin, and avicularin in apple flesh samples of the ‘Galaval’ cultivar grown on rootstocks P 67 and 62-396-B10^®^ compared to the levels found by Kviklys et al. in 2014 when testing apple samples of the ‘Ligol’ cultivar grown on rootstocks P 67 and B.396 [22].

**Figure 5:**
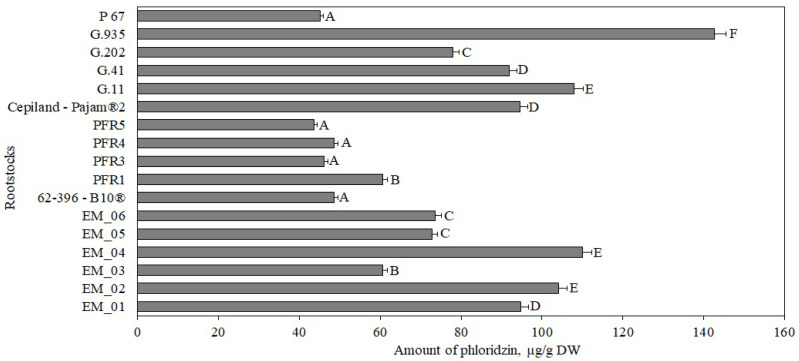
Influence of rootstocks on the quantitative composition of phloridzin in apple flesh samples. The means followed by different letters are significantly different at *p* < 0.05.

Our study showed that the amount of phloridzin in apple samples of the ‘Galaval’ cultivar ranged from 44 ± 4 μg/g to 143 ± 15 μg/g (Figure 5). The statistically significant largest amount of phloridzin (143 ± 15 μg/g) was found in flesh samples of apples grown on the strong-dwarf rootstock G.935, and the smallest amount (44 ± 4 μg/g) was found in flesh samples of apples grown on the dwarf rootstock PFR5 (Figure 5). In our study, apple flesh samples of the ‘Galaval’ cultivar grown on rootstocks had higher levels of phloridzin compared to the amount found in apple flesh samples of the ‘Brookfield Gala’ cultivar (33 μg/g) [38].

The estimation of the influence of rootstocks of different growth groups and origin on the content of phenolic acid and flavonoids in apple flesh of the ‘Galaval’ cultivar showed that the growth group and origin of the rootstocks did not have any significant effect on the accumulation of phenolic compounds. Interestingly, it showed that the content of phenolic compounds depended more on the rootstock genotype.

### 3.2. Influence of Rootstocks on the Phenolic Content in Apple Peel Samples

According to scientific literature, the qualitative and quantitative composition of biologically active compounds varies depending on the parts of the apple fruit, i.e., peel and flesh [44]. The highest content of lipophilic biologically active compounds is accumulated in the cuticle wax layer of apple peel [45,46]. A review of scientific literature did not yield any studies describing the influence of rootstocks on the content of phenolic compounds in different parts of the fruit, i.e., in the peel and flesh. We determined how rootstocks of different origin and vigor affect the phenolic compound profiles in apple peel and obtained results that we compared with results found in apple flesh.

**Figure 6:**
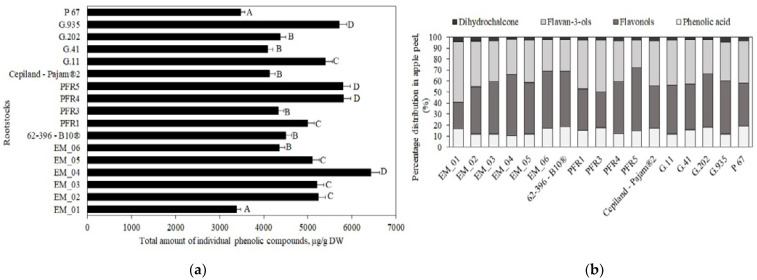
Influence of rootstocks on the quantitative composition of phenolic compounds: (**a**) Total variation of phenolic compounds in apple peel samples; (**b**) percentage distribution of phenolic compounds in apple peel samples. The means followed by different letters are significantly different at *p* < 0.05.

Our study showed that, depending on the rootstocks, the total amount of phenolic compounds in apple peel samples of the ‘Galaval’ cultivar ranged from 3380 ± 169 μg/g to 6434 ± 322 μg/g (Figure 6, Panel a). The highest total amount of phenolic compounds (6434 ± 322 μg/g) was found in peel samples of apples grown on the super dwarf rootstock EM_04, and the smallest amount (3380 ± 169 μg/g) was found in peel samples of apples grown on the semi-vigorous rootstock EM_01 from the same breeding program in Great Britain (Figure 6, Panel a). Mainla et al. found that the dwarfing effect of B.396 and M.26 rootstocks might have had a beneficial influence on the concentrations of polyphenols because canopies with weaker shoot growth provided better light conditions for fruits [19]. This could be attributed to the genetic adjustment of the metabolism between rootstock and scion cultivars, which causes higher levels of metabolic stress.

Our study showed that, depending on the rootstock, the content of phenolic compounds in apple peel samples was twice as high as that in apple flesh samples. Milosevic et al. found that apple peel samples of the ‘Red Chief^®^ Camspur’ cultivar grown on the rootstock M.9 T337 contained higher levels of phenolic acids and flavonoids than the apple flesh samples [47]. Slatnar et al. found that apple peel samples of the ‘Ligol’ cultivar grown on rootstocks M.26 and P 60 contained higher levels of phenolic compounds than the apple flesh samples [40]. The evaluation of the dynamics of the composition of phenolic compounds between the individual parts of ‘Galaval’ apples showed that in apple peel samples, differently from apple flesh samples, flavonols predominated, their percentage distribution ranging from 24% to 58% of the total amount of the identified and quantified phenolic compounds (Figure 6, Panel b). Geana et al. found that flavonols and anthocyanins were usually found in apple peel, while flavan-3-ols, dihydrochalcones, and hydroxycinnamic acids were the major polyphenol groups found in apple flesh [48].

**Figure 7:**
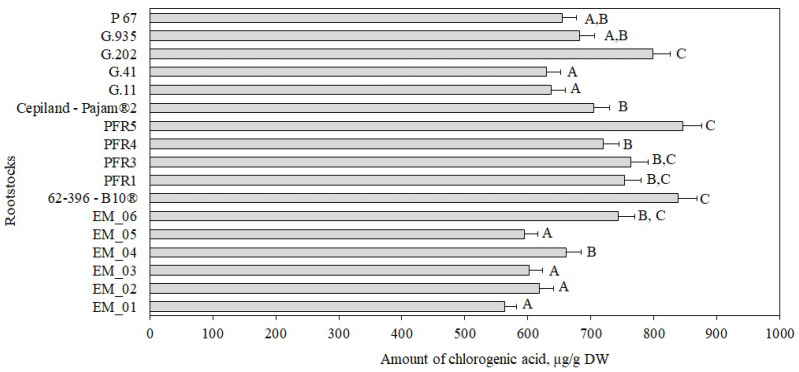
Influence of rootstocks on the quantitative composition of chlorogenic acid in apple peel samples. The means followed by different letters are significantly different at *p* < 0.05.

In our study, the largest amount of chlorogenic acid (845 ± 21 μg/g) found in peel samples of apples grown on the rootstock PFR5 did not differ significantly statistically from that found in peel samples of apples grown on rootstocks G.202, PFR1, PFR3, EM_06, or 62-396-B10^®^ (Figure 7). Meanwhile, the smallest amount of chlorogenic acid (563 ± 14 μg/g) was found in peel samples of apples grown on the semi-vigorous rootstock EM_01 (Figure 7). We found that, depending on the rootstock, apple peel samples accumulated 8.6–10.5 times more chlorogenic acid than apple flesh samples did.

Mainla et al. found that the amount of chlorogenic acid in apple peel samples of the ‘Talvenauding’ cultivar grown in Estonia on rootstocks B.396 and M.26 ranged from 51 μg/g to 130 μg/g [19]. We found that apple peel samples of the ‘Galaval’ cultivar, which is cultivated in Lithuania, when grown on the rootstock 62-396-B10^®^, contained a larger amount of chlorogenic acid (839 ± 23 μg/g) compared to that found in apple peel samples of the ‘Talvenauding’ cultivar grown in Estonia.

**Figure 8:**
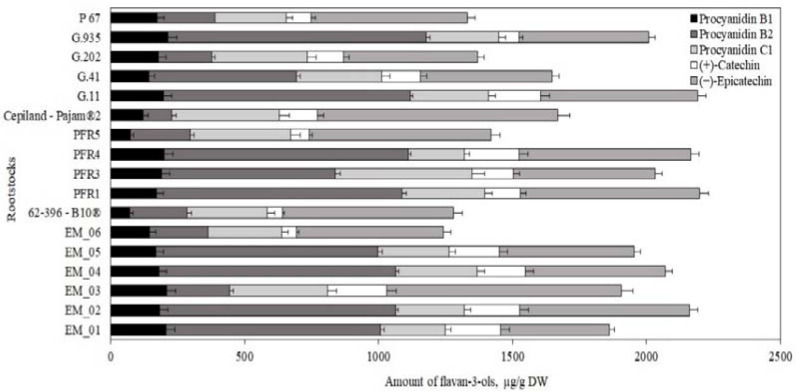
Influence of rootstocks on the quantitative composition of flavan-3-ols in apple peel samples.

The total amount of flavan-3-ols in apple peel samples varied almost twice depending on the rootstock, from 1243 ± 62 μg/g to 2200 ± 109 μg/g (Figure 8). The highest total amount of flavan-3-ols (2200 ± 109 μg/g) was found in peel samples of apples grown on the semi-dwarf rootstock PFR1, and the smallest amount (1243 ± 62 μg/g) was found in peel samples of apples grown on the semi-dwarf rootstock EM_06 (Figure 8). Slatnar et al. studied apple samples of the ‘Ligol’ cultivar grown on rootstocks M.9, M.26, and P 60, and found that flavan-3-ol content ranged from 280 μg/g to 416 μg/g [40]. In our study, apple peel samples of the ‘Galaval’ cultivar grown on rootstocks had larger amounts of flavan-3-ol on compared to the amount (702 μg/g) found in apple peel samples of the ‘Brookfield Gala’ cultivar [38].

Apple peel samples were found to contain 1.5 times higher (−)-epicatechin and 1.2 times higher (+)-catechin levels than apple flesh samples did. The largest amount of (−)-epicatechin (897 ± 26 μg/g) was found in peel samples of apples grown on the strong-dwarf rootstock Cepiland-Pajam^®^2, while the smallest amount (406 ± 20 μg/g) was found in peel samples of apples grown on the semi-vigorous rootstock EM_01 (Figure 8). The largest amount of (+)-catechin (221 ± 12 μg/g) was found in peel samples of apples grown on the small-dwarf rootstock EM_03, while the smallest amount (55 ± 3 μg/g) was found in peel samples of apples grown on the semi-dwarf rootstock EM_06 (Figure 8). Mainla et al. examined apple peel samples of the ‘Talvenauding’ cultivar grown on rootstocks B.396 and M.26 and found that the amount of (+)-catechin ranged from 91 μg/g to 143 μg/g [19]. In our study, we found a smaller amount of (+)-catechin (55 ± 3 μg/g) in apple peel samples of the ‘Galaval’ cultivar grown on the rootstock 62-396-B10^®^ compared to that found in apple peel samples of the ‘Talvenauding’ cultivar grown on the rootstock B.396 [19]. In a study by Loncaric et al., the amount of (−)-epicatechin (414 μg/g) found in apple peel samples of the ‘Gala’ cultivar was lower compared to that we found in apple peel samples of the ‘Galaval’ cultivar [49].

The amount of procyanidins in apple peel samples varied depending on the rootstock. The largest amounts of procyanidin B2 and procyanidin B1 (respectively, 964 ± 48 μg/g and 214 ± 11 μg/g) were found in peel samples of apples grown on the strong-dwarf rootstock G.935 (Figure 8). The largest amount of procyanidin C1 (512 ± 26 μg/g) was found in peel samples of apples grown on the semi-dwarf rootstock PFR3 (Figure 8). The amounts of procyanidin B1 and procyanidin B2 in apple peel samples of the ‘Gala’ cultivar found in a study by Loncaric et al. (respectively, 183 μg/g and 619 μg/g) were lower compared to those we found in apple peel samples of the ‘Galaval’ cultivar grown on rootstocks [49]. No significant difference in flavan-3-ol content was found between apple flesh and apple peel samples. Hagen et al. found that (−)-epicatechin, procyanidins, phloridzin, and chlorogenic acid may be detected in both apple peel and apple flesh samples [50].

**Figure 9:**
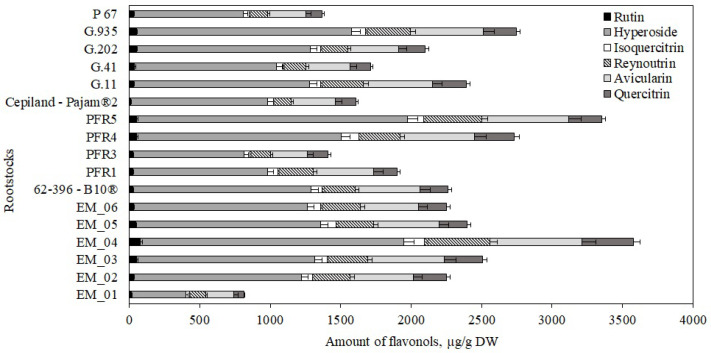
Influence of rootstocks on the quantitative composition of flavonols in apple peel samples.

The total flavonol content in apple peel samples, depending on the rootstock, was found to be by 1.9−4.1 times higher than that found in apple flesh samples. The largest amount of flavonols (3580 ± 179 μg/g) was found in peel samples of apples grown on the super dwarf rootstock EM_04, while the smallest amount 812 ± 34 μg/g was found in peel samples of apples grown on the semi-vigorous rootstock EM_01 (Figure 9). Slatnar et al. in their study found that the amount of flavonols in the peel samples of the ‘Ligol’ apple cultivar ranged from 325 μg/g to 1086 μg/g, depending on the rootstocks (M.9, M.26, or P 60) [40].

We determined the dynamics of the variation in the content of individual flavonols in apple peel samples depending on the rootstock. The largest amount of hyperoside (1920 ± 96 μg/g) was found in peel samples of apples grown on the dwarf rootstock PFR5, while the lowest (391 ± 15 μg/g) was found in peel samples of apples grown on the semi-vigorous rootstock EM_01 (Figure 9). The largest amounts of avicularin, reynoutrin, quercitrin, isoquercitrin, and rutin (accordingly, 656 ± 33 μg/g, 462 ± 20 μg/g, 366 ± 19 μg/g, 150 ± 8 μg/g, and 79 ± 3 μg/g) were found in peel samples of apples grown on the super dwarf rootstock EM_04 (Figure 9). Meanwhile, the lowest levels of avicularin, reynoutrin, quercitrin, and isoquercitrin (accordingly, 200 ± 10 μg/g, 113 ± 6 μg/g, 69 ± 4 μg/g, and 27 ± 1 μg/g) were found in apple peel samples grown on the semi-vigorous rootstock EM_01 (Figure 9). The smallest amount of rutin (12 ± 1 μg/g) was found in apple peel samples grown on the strong-dwarf rootstock Cepiland-Pajam^®^2 (Figure 9). Mainla et al. in their study found that the amount of hyperoside in apple peel samples of the ‘Talvenauding’ cultivar grown on rootstocks B.396 and M.26 ranged from 163 μg/g to 463 μg/g [19]. In our study, the amount of hyperoside (1265 ± 64 μg/g) in apple peel samples of the ‘Galaval’ cultivar grown on the rootstock 62-396-B10^®^ was higher than that found by Mainla et al.

**Figure 10:**
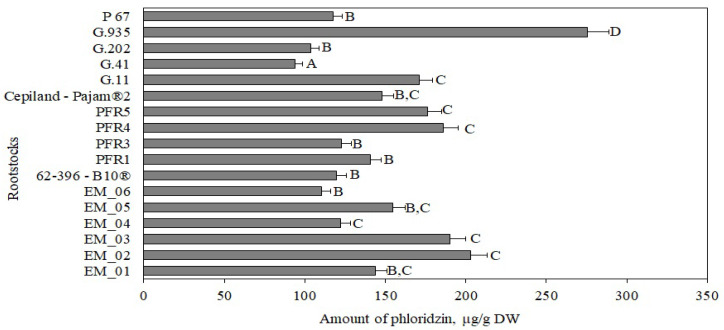
Influence of rootstocks on the quantitative composition of phloridzin in apple peel samples. The means followed by different letters are significantly different at *p* < 0.05.

The amount of phloridzin in apple peel samples, depending on the rootstock, ranged from 94 ± 5 μg/g to 275 ± 15 μg/g (Figure 10). The statistically significantly largest amount of phloridzin (275 ± 15 μg/g) was found in peel samples of apples grown on the strong-dwarf rootstock G.935, and the lowest (94 ± 5 μg/g) was found in peel samples of apples grown on the strong-dwarf rootstock G.41 (Figure 10).

Our study showed that, depending on the rootstock, the amount of phloridzin in apple peel samples was almost twice as high as in apple flesh samples. Slatnar et al. found that the amount of dihydrochalcone in apple peel samples of the ‘Ligol’ cultivar grown on rootstocks M.9, M.26, and P 60 ranged from 38 μg/g to 59 μg/g [40]. Loncarinc et al. found that the amount of phloridzin in apple peel samples of the ‘Gala’ cultivar reached 73 μg/g [49].

The rootstock–scion combination influenced the content of phenolic compounds in the apple peel. We determined that depending on rootstocks in apple peel accumulated 1.9–2.6 times higher total amount phenolic compounds compared to content estimated in apple flesh. In apple peel, the greatest variation was assessed for the total flavonol content (by 4.4 times), and the total flavan-3-ol content varied the least (by 1.8 times) collated to amount found in apple flesh. Mainla et al. showed that the apple peel samples with higher content of total phenolic compounds also had a higher content of flavonols and (+)-catechin [19]. Hagen et al. found that anthocyanins and quercetin glycosides existed exclusively in apple peels, whereas (−)-epicatechin, procyanidins, phloridzin, and chlorogenic acid were found in both peel and flesh of the apples [50]. Wojdyło et al. also demonstrated that genotypes with the highest phenolic concentration had simultaneously high contents of flavonols [51].

### 3.3. Hierarchical Cluster Analysis of Phenolic Compounds

**Figure 11:**
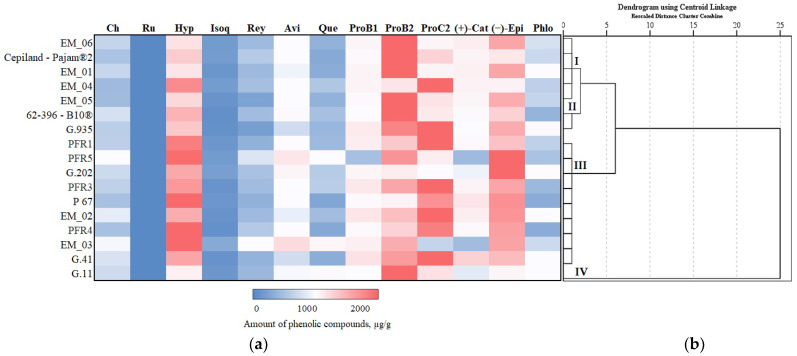
Influence of rootstocks on the quantitative composition of phenolic compounds: (**a**) Heatmap reveals the variation in the quantitative composition of individual phenolic compounds in apple flesh samples; (**b**) the dendrogram illustrates variation in the quantitative composition of phenolic compounds in apple flesh samples.

**Figure 12:**
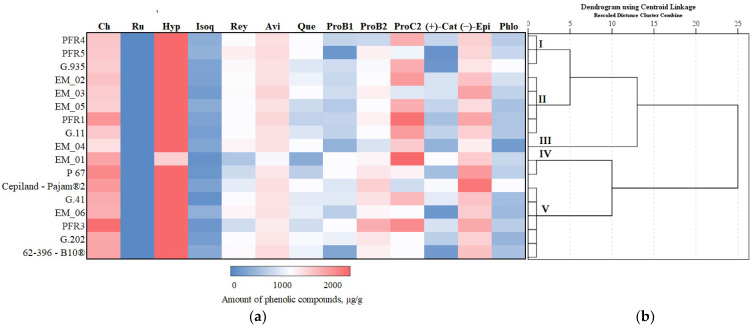
Influence of rootstocks on the quantitative composition of phenolic compounds: (**a**) Heatmap reveals the variation in the quantitative composition of individual phenolic compounds in apple peel samples; (**b**) the dendrogram illustrates variation in the quantitative composition of phenolic compounds in apple peel samples.

We systematized research data on the influence of apple rootstocks on the qualitative and quantitative composition of individual phenolic compounds. Depending on the rootstock, a hierarchical cluster analysis of phenolic compounds of apple flesh and apple peel was performed, the results of which are presented in Figure 11 and Figure 12.

In apple flesh samples, the total amount of phenolic compounds was divided into four clusters depending on the rootstock (Figure 11). Cluster I, where average amounts of total phenolic compounds in apple flesh samples were detected, included almost all rootstocks produced in the United Kingdom (EM_01, EM_04, EM_05, and EM_06) and the strong-dwarf rootstock Cepiland-Pajam^®^2 produced in France. Cluster II with lower-than-average total phenolic compounds in apple flesh samples included the dwarf rootstock 62-396-B10^®^ from Russia and a strong-dwarf rootstock G.935 originating from the USA. Cluster III, which had the lowest total phenolic levels in apple flesh samples, included all rootstocks produced in New Zealand (PFR1, PFR3, PFR4, and PFR5), small-dwarf rootstocks of higher growth produced in the United Kingdom (EM_02 and EM_03), rootstocks G.41 and G.202 originating from the USA, and the rootstock P 67 originating from Poland. The strong-dwarf rootstock G.11 produced in the USA was assigned to Cluster IV, which had the highest total amount of phenolic compounds in apple flesh samples (Figure 11).

In apple peel samples, the total amount of phenolic compounds was divided into five clusters depending on the rootstock (Figure 12). Cluster I, which contained less than the highest total amount of phenolic compounds in apple peel samples, included rootstocks PER4, PER5, and G.935. Cluster II, where higher than average total amounts of phenolic compounds in apple peel samples were found, included rootstocks EM_02, EM_03, and EM_05 from the United Kingdom, PER1 from New Zealand, and G.11 from the USA. Cluster III with the highest total content of phenolic compounds in apple peel samples included the super-dwarf rootstock EM_04 from the United Kingdom. Cluster IV, which had the lowest total amount of phenolic compounds in apple peel samples, included the semi-vigorous rootstock EM_01 and the dwarf rootstock P 67. Cluster V with average total amounts of phenolic compounds in apple peel samples included rootstocks Cepiland-Pajam^®^2, 62-396-B10^®^, G.41, G.202, EM_06, and PER3 (Figure 12).

According to scientific literature, the qualitative and quantitative composition of phenolic compounds in apples depends on various factors: cultivar properties, fruit maturity, weather conditions of the harvesting season, processing, agricultural conditions, crop load, development of infection, fruit position within the canopy, and geographic location [52,53]. On the other hand, considering factors influencing apple quality, the list also includes rootstocks [19]. The cluster analysis showed that the content of phenolic compounds was not found to correlate with the growth of the rootstocks. That was not evident in our previous study either, where we tested rootstocks from three different vigor classes [22]. However, the total amount of phenolic compounds depended more on the rootstock genotype. Furthermore, we found that rootstocks influenced the content of phenolic compounds in different parts of the apples, i.e., in apple flesh and peel. In apple flesh the whole group of rootstocks produced in New Zealand (PFR series rootstocks) induced lower amounts, while the majority of rootstocks produced in Great Britain (EM series rootstocks) induced significantly higher total amounts phenolic compounds, and G series rootstocks from the USA exhibited a much greater variation. Our study showed that the amount of chlorogenic acid in apple flesh samples varied depending on the rootstock genotype, but the vigor of the rootstock did not affect the quantitative composition of chlorogenic acid. Rootstocks produced in Great Britain induced higher levels of (+)-catechin and (−)-epicatechin, hyperoside, avicularin, reynoutrin, and isoquercitrin. The highest levels of phloridzin, quercitrin, and rutin were found in apple flesh grown on rootstocks produced in the USA and Russia, respectively. The highest total amount of phenolic compounds was found in peel samples of apples grown on the rootstock originating from the Great Britain. The highest chlorogenic acid content was found in peel samples of apples grown on the rootstock originating from New Zealand. Rootstocks originating from New Zealand and the United Kingdom also induced the highest levels of, respectively, flavan-3-ols and flavonols in apple peel samples. The results of the analysis confirmed the ability of these rootstocks to accumulate larger amounts of phenolic compounds.

The results of this study demonstrated that the rootstocks altered the dynamics of biochemical characteristics. From a health point of view, the concentration of polyphenols is considered as an important constituent of apple quality. The influence of the rootstock should also be taken into account as an important factor influencing the concentration of phenolic compounds. A proper matching of the cultivar and the rootstock can program the fruit tree to produce larger amounts of higher-quality, antioxidant-rich, and high-nutrition-value fruit.

### 3.4. Influence of Rootstocks on the Antioxidant Activity of Apple Flesh and Peel Samples

Phenolic compounds are one of the main groups of biologically active compounds that determine the antioxidant activity of fruit samples [2,54]. The antioxidant activity of phenolic compounds is determined by the hydroxyl groups and their redox properties, due to which they act as reducing agents, hydrogen ion donors, singlet oxygen quenchers, or metal ion chelators [54]. Oxidative stress is associated with a variety of chronic and neurodegenerative diseases [55]. It is advisable to include fruits and vegetables with the strongest antioxidant activity in the diet. Scientific literature provides fragmentary data on the effect of rootstocks on the antioxidant activity of apple samples. The antioxidant activity of apple flesh and peel samples of the ‘Galaval’ cultivar grown on rootstocks was evaluated by using two different methods in vitro. The antiradical activity of apple flesh and peel extracts was evaluated by using the DPPH free radical scavenging method, and the reducing activity was evaluated by applying the FRAP method in vitro.

**Figure 13:**
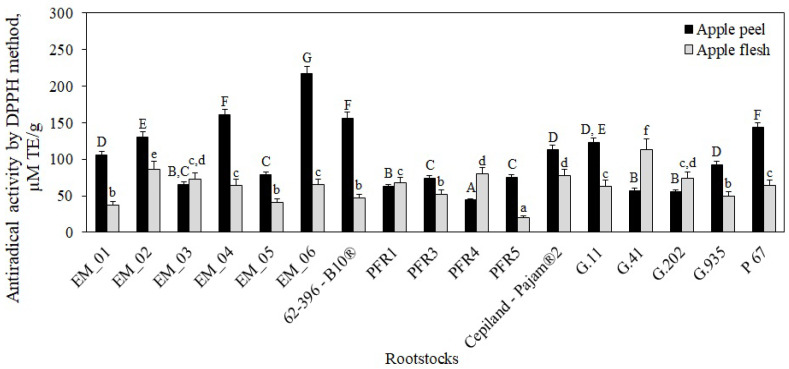
Influence of rootstocks on antiradical activity of apple flesh and apple peel extracts determined by the DPPH free radical scavenging method. The means followed by different uppercase and lowercase letters are significantly different at *p* < 0.05.

The antiradical activity of apple flesh samples ranged from 20 ± 1 μM TE/g to 114 ± 15 μM TE/g, depending on the rootstock (Figure 13). The strongest antiradical activity (114 ± 15 μM TE/g) was found in flesh samples of apples grown on the strong-dwarf rootstock G.41, and the weakest (20 ± 1 μM TE/g) was found in flesh samples of apples grown on the dwarf rootstock PFR5 (*p* < 0.05) (Figure 13).

Depending on the rootstock, the antiradical activity of apple peel samples ranged from 44 ± 2 μM TE/g to 217 ± 10 μM TE/g (Figure 13). Statistically significantly, the strongest antiradical activity (217 ± 10 μM TE/g) was detected in peel extracts of apples grown on the semi-dwarf rootstock EM_06, and the weakest activity (44 ± 2 μM TE/g) was found in peel extracts of apples grown on the semi-dwarf rootstock PFR4 (*p* < 0.05) (Figure 13). Peel extracts of apples grown on rootstocks EM_03, PFR1, PFR4, G.41, and G.202 showed weaker antiradical activity compared to that detected in apple flesh extracts. Lauzike et al. found that sample extracts of apples grown on the super-dwarf rootstock P 22 had by 33–44% lower antiradical activity compared to that found in sample extracts of apples grown on the dwarf rootstock P 60 [30]. Milocevic et al. found that flesh and peel extracts of apples grown on rootstock M.9 T337 had stronger antioxidant activity compared to flesh and peel samples of apples grown on the more vigorous rootstocks M.4 and MM.106 [47]. The findings obtained by these researchers confirm the results of our study showing that the antioxidant activity varied between apple peel and flesh and between rootstocks of different growth groups and origins.

**Figure 14:**
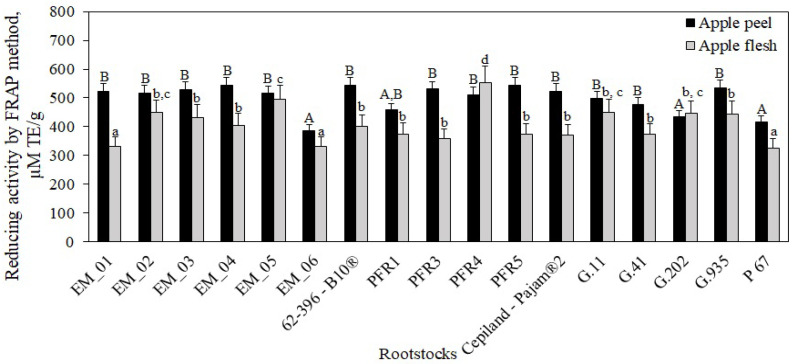
Influence of rootstocks on the reducing activity of apple flesh and apple peel extracts determined by the FRAP method. The means followed by different uppercase and lowercase letters are significantly different at *p* < 0.05.

Our study showed that the reducing activity of apple flesh samples determined by the FRAP method varied from 326 ± 17 μM TE/g to 554 ± 22 μM TE/g, depending on the rootstock (Figure 14). The strongest reducing activity (554 ± 22 μM TE/g) was found in flesh extracts of apples grown on the semi-dwarf rootstock PFR4 (*p* < 0.05), and the weakest (326 ± 17 μM TE/g) was found in flesh extracts of apples grown on the dwarf rootstock P 67, which was not statistically significantly different from the activity found in flesh extracts of apples grown on rootstocks EM_01 or EM_06 (Figure 14). Apple flesh samples did not show a statistically significant difference in reducing activity between all rootstocks produced in the USA, almost all rootstocks produced in the United Kingdom and New Zealand, and those produced in France and Russia.

In our study, we found that the reducing activity of apple peel sample extracts determined by the FRAP method varied from 387 ± 19 μM TE/g to 545 ± 28 μM TE/g, depending on the rootstock (Figure 14). The strongest reducing activity (545 ± 28 μM TE/g) was found in peel extracts of apples grown on the dwarf rootstock PFR5, although it did not differ significantly from that found in peel extracts of apples grown on most other rootstocks (*p* > 0.05) (Figure 14). The weakest reduction activity (387 ± 19 μM TE/g) was found in peel extracts of apples grown on the semi-dwarf rootstock EM_06, and it did not differ statistically significantly from reduction activity found in peel extracts of apples grown on rootstocks PFR1, G.202., and P67 (Figure 14). According to the results obtained by Xu et al., the reducing activity evaluated by the FRAP method in apple flesh samples of the ‘Gale Gala’ cultivar was 37 μM TE/g, and in peel samples 76 μM TE/g, which is below the reducing activity in apple peel and flesh samples of the ‘Galaval’ cultivar found in our study [56]. In our study, we found that peel extracts of apples grown on rootstocks PFR4 and G.202 had weaker reducing activity compared to that found in apple flesh samples. This trend was also found when evaluating the antiradical activity in relation to these rootstocks by using the DPPH method. Milosevic et al. found that the dwarf rootstock ‘M.9 T337’ induced a higher antioxidant power than semi-dwarf rootstocks M.4 or MM.106. Studies showed that M.4 induced the lowest antioxidant activity in apple flesh, whereas both M.4 and MM.106 rootstocks induced statistically similar antioxidant capacity in apple peel [47]. The results of the study by Milocevic et al. confirm our findings indicating that the antioxidant activity in apple samples varied depending on the rootstock. Eberhardt et al. found that apple peel contained more antioxidant compounds, especially quercetin, so the peel might have higher antioxidant activity and higher bioactivity than the flesh [57]. Li et al. provided evidence that apple peel was directly exposed to (a) biotic stress, which resulted in a faster synthesis of phenolic compounds, and stronger antioxidant activity of apple peel extracts compared to apple flesh samples [5]. Rootstocks improve tolerance to various environment stresses by producing different types of antioxidants that scavenge or detoxify reactive oxygen species. These antioxidants are health-promoting phytochemicals, and their accumulation increases the health value and quality of fruits [58,59]. Scientific literature provides data on the multifaceted biological effects of individual phenolic compounds. Chlorogenic acid accumulated in apples have strong antioxidant effect [60]. Feng et al. estimated that chlorogenic acid has stronger antioxidant properties than quercetin, gallic acid, or α-tocopherol, but its antioxidant activity is weaker than that of rutin [61]. Flavan-3-ol group compounds, such as procyanidin B1, have been shown to have anti-Alzheimer’s effects [62], (−)-epicatechin offers benefits regarding renal alterations associated with inflammatory or metabolic diseases and increased muscle growth and strength [48,63], and (+)-catechin has anti-obesity effects [64]. Procyanidin B1, (+)-catechin, and (−)-epicatechin are the main compounds in apples responsible for reducing cholesterol levels [65]. Flavonols are potent antioxidants with anti-inflammatory, anticancer, anticoagulant, antiallergic, and antiviral effects [66]. Phloridzin can inhibit lipid peroxidation and has been proposed as an antihyperglycemic and antihyperlipidemic agent in diabetes and a potential therapeutic agent in obesity [2,38]. Conducted studies have demonstrated that phenolic compounds have photoprotective effects and reduced premature aging [67,68].

Despite wide use of in vitro antioxidant assays to distinguish which antioxidants and foods are most effective, the assays have some limitations. Most antioxidants are poorly absorbed or are rapidly conjugated and eliminated in the urine, so circulating phenolic compound concentrations reach small quantities. Phenolic compounds bind easily to proteins in cells and foods, so they may be inactivated. Therefore, content of phenolic compounds used in in vitro assays are orders of magnitude higher than would ever be found in in vivo assays. In general, in vitro assays have a weak reproducibility of the physiological environment conditions compared to the in vivo assays [69]. In future research, it is appropriate to conduct new antioxidant assays with clearly identifiable reaction mechanisms, fully tested reaction conditions, and kinetics.

## 4. Conclusions

The rootstock–scion combination had a significant effect on the qualitative and quantitative composition and in vitro antioxidant activity of phenolic compounds in apple flesh and apple peel samples. The results obtained did not depend on the growth class of the rootstock but were strongly influenced by the rootstock genotype: rootstocks produced in New Zealand (PFR series rootstocks) induced smaller total amounts of phenolic compounds in apple flesh, while the rootstock series from Great Britain (EM series rootstocks) induced a consistently higher content. The highest total content of phenolic compounds was found in peel samples of apples grown on the super dwarf rootstock EM_04 from the United Kingdom. Meanwhile, peel samples of apples grown on the semi-vigorous rootstock EM_01 of the same breeding program had the lowest content of phenolic compounds.

The rootstock–scion combination also determined the biochemical value of the fruit. Depending on the rootstock, the total content of phenolic compounds in apple flesh of the ‘Galaval’ cultivar could vary by 2.9 times, and in the peel by up to 90%. The genotype of the rootstock resulted in the highest variation in total flavan-3-ol content in apple flesh, by as much as 4.3 times, while the total content of flavonols varied by 2.1 times. In apple peel, on the contrary, the greatest variation was recorded for the total flavonol content (by 4.4 times), and the total flavan-3-ol content varied the least (by 1.8 times). The greatest variation of individual compounds in the fruit flesh depending on the rootstock (by 18.9 times) was recorded for procyanidin B2. The most stable compound in the fruit flesh was hyperoside, with a variation of 1.9 times. In apple peel, the rootstock choice resulted in a 4.9-fold variation in total hyperoside content. The most stable compound was chlorogenic acid, which varied by only 50% depending on the rootstock.

We evaluated the effect of rootstocks on the antioxidant activity of apple flesh and peel samples in vitro. Antioxidant activity did not depend on the growth class of the rootstocks but depended on their genotypes. Flesh and peel samples of apples grown on the rootstocks produced in the USA (G series rootstocks) and the United Kingdom (EM series rootstocks) showed the strongest antiradical activity determined by the DPPH method. Meanwhile, flesh and peel samples of apples grown on the rootstocks produced in New Zealand (PFR series rootstocks) showed the strongest reducing activity as assessed by the FRAP method.

A proper match of a cultivar and a rootstock can program a fruit tree to grow larger amounts of higher-quality, antioxidant-rich, and high-nutrition-value fruit. Such combinations could be used when designing modern gardens, and the fruit would be essential for practical and fundamental medicine and in the production of novel supplements, foods, or other products.

## Figures and Tables

**Table 1:** Origin and growth characteristics of apple rootstocks.

No.	Rootstock	Country of Origin	Vigor According to Breeders	Actual Vigor
1.	EM_01	UK	Semi-dwarf	Semi-vigorous
2.	EM_02	UK	Dwarf	Small dwarf
3.	EM_03	UK	Dwarf	Small dwarf
4.	EM_04	UK	Dwarf	Super dwarf
5.	EM_05	UK	Dwarf	Small dwarf
6.	EM_06	UK	Dwarf	Semi-dwarf
7.	62-396-B10^®^	Russia	Dwarf	Dwarf
8.	PFR1	New Zealand	Semi-dwarf	Semi-dwarf
9.	PFR3	New Zealand	Semi-dwarf	Semi-dwarf
10.	PFR4	New Zealand	Dwarf	Semi-dwarf
11.	PFR5	New Zealand	Dwarf	Dwarf
12.	Cepiland-Pajam^®^2	France	Dwarf	Strong-dwarf
13.	G.11	USA	Dwarf	Strong-dwarf
14.	G.41	USA	Dwarf	Strong-dwarf
15.	G.202	USA	Semi-dwarf	Semi-dwarf
16.	G.935	USA	Semi-dwarf	Strong-dwarf
17.	P 67	Poland	Dwarf	Dwarf

## Data Availability

All datasets generated for this study are included in the article.
